# Supplementary material for: Rapid protection from COVID-19 in nonhuman primates vaccinated intramuscularly but not intranasally with a single dose of a recombinant vaccine
Source: bioRxiv. 2021 Jan 19:2021.01.19.426885. Preprint. [Version 1] doi: 10.1101/2021.01.19.426885 (PMC7836117; doi:10.1101/2021.01.19.426885)
Supplement: 1 [file NIHPP2021.01.19.426885-supplement-1.pdf]

bioRxiv preprint doi: https://doi.org/10.1101/2021.01.19.426885; this version posted January 19, 2021. The copyright holder for this preprint (which was not certified by peer review) is the author/funder. This article is a US Government work. It is not subject to copyright under 17 USC 105 and is also made available for use under a CC0 license.

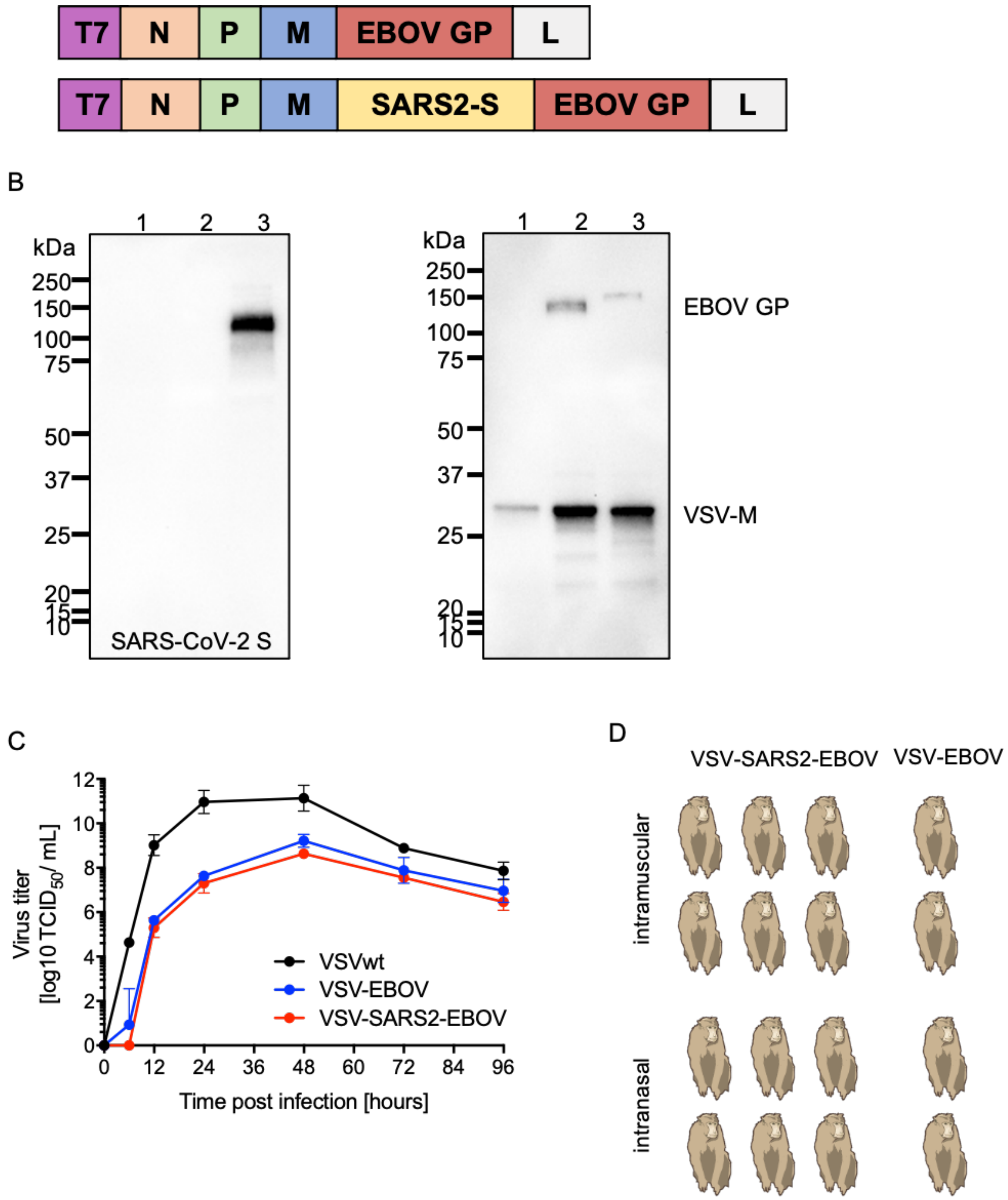


**Figure S1. Schematic and characterization of VSV-based vaccines. (A)** Schematic illustrating vaccine vector design. T7 promotor; N nucleoprotein; P phosphoprotein; M matrix protein; EBOV GP Ebola virus glycoprotein; L RNA-dependent RNA polymerase; SARS2-S SARS-CoV-2 S. **(B)** Western blot analysis of cell supernatant samples containing VSV vaccines probed for SARS-CoV-2 S (left), VSV M (middle) or EBOV GP (right). 1 VSV wildtype (VSVwt); 2 VSV- EBOV; 3 VSV- SARS2-EBOV. **(C)** Viral growth kinetics on VeroE6 cells. Geometric mean and SD are depicted. Results are not statistically significant. **(D)** Schematic outline of the rhesus macaque study.

bioRxiv preprint doi: https://doi.org/10.1101/2021.01.19.426885; this version posted January 19, 2021. The copyright holder for this preprint (which was not certified by peer review) is the author/funder. This article is a US Government work. It is not subject to copyright under 17 USC 105 and is also made available for use under a CC0 license.

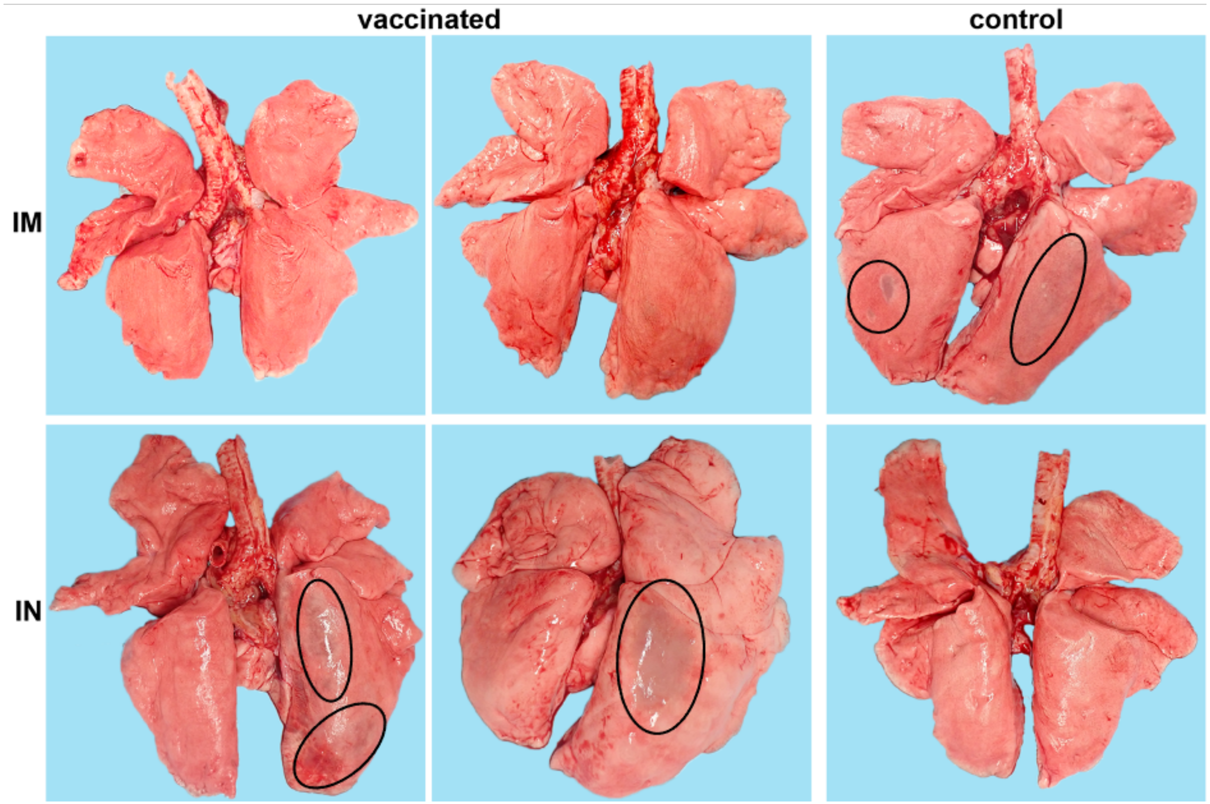


**Figure S2. Lung gross pathology of NHPs.** Representative pictures of NHP lungs with lesions on day 7 are shown. IM intramuscular vaccination; IN intranasal vaccination. Lesions are circled.

bioRxiv preprint doi: https://doi.org/10.1101/2021.01.19.426885; this version posted January 19, 2021. The copyright holder for this preprint (which was not certified by peer review) is the author/funder. This article is a US Government work. It is not subject to copyright under 17 USC 105 and is also made available for use under a CC0 license.

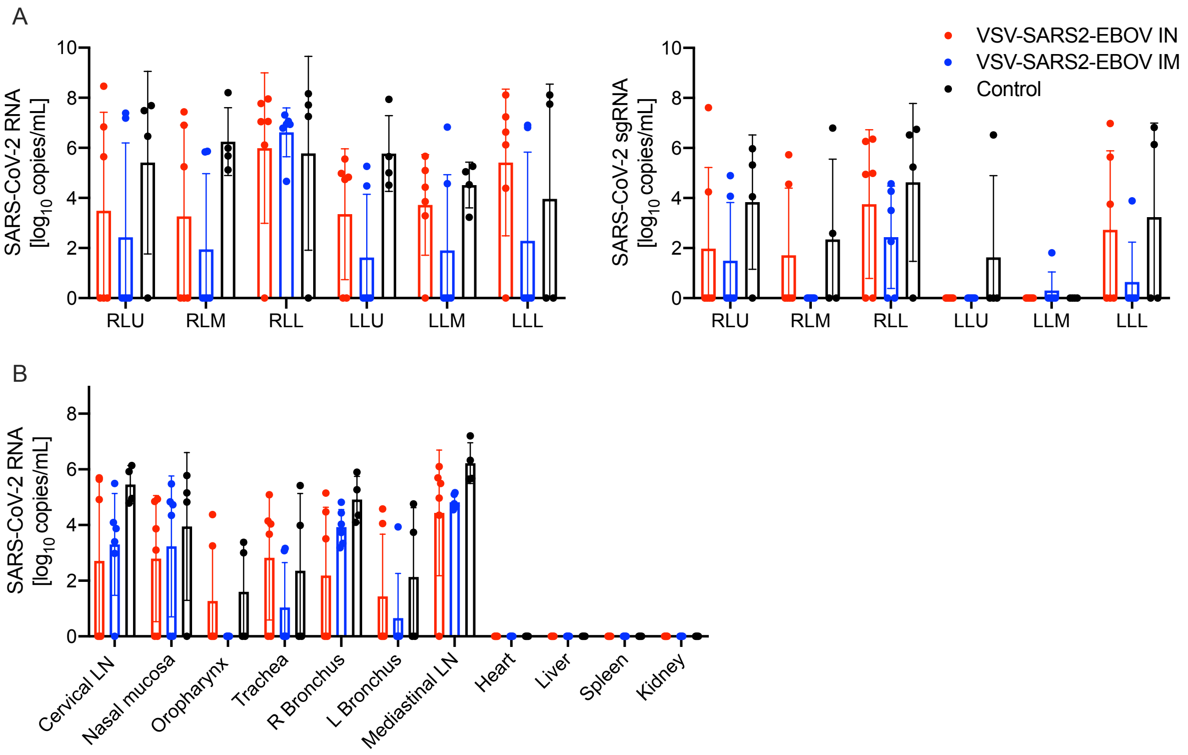


**Figure S3. Virus load in NHP tissue samples on day 7. (A)** Total SARS-CoV-2-specific RNA (left panel) and subgenomic (sg) RNA (right panel) in lung samples collected from NHPs. RLU right lobe upper; RLM right lobe middle; RLL right lobe lower; LLU left lobe upper; LLM left lobe middle; LLL left lobe lower. **(B)** Total SARS-CoV-2-specific RNA in tissue samples (right panel) from NHPs. LN lymph node; R right; L left.

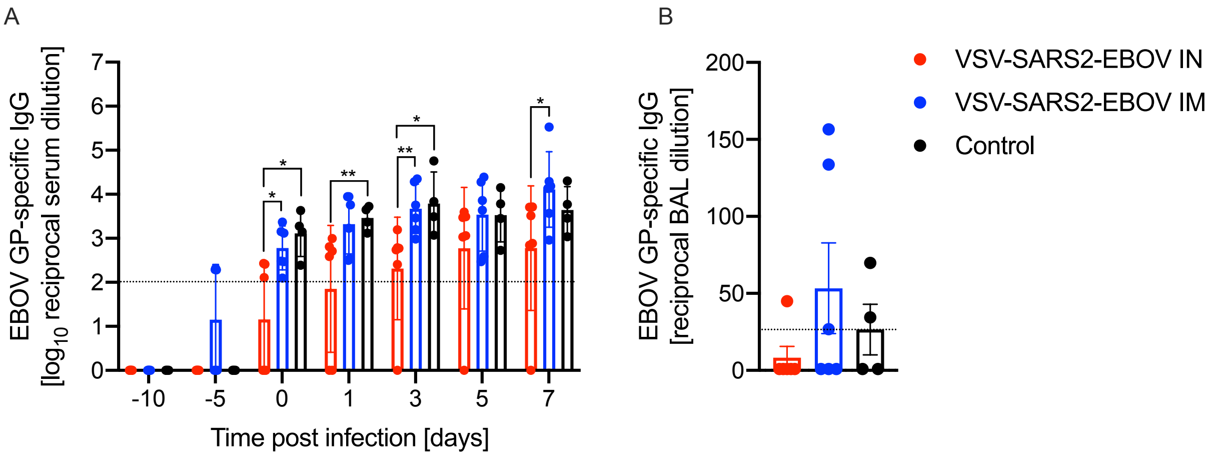


**Figure S4. EBOV GP-specific antibodies in NHPs after vaccination and challenge.** Bronchoaveolar lavage (BAL) samples collected on day 3 and serum samples collected throughout the study were analyzed by ELISA for EBOV GP-specific IgG. Statistical significance is indicated.

bioRxiv preprint doi: https://doi.org/10.1101/2021.01.19.426885; this version posted January 19, 2021. The copyright holder for this preprint (which was not certified by peer review) is the author/funder. This article is a US Government work. It is not subject to copyright under 17 USC 105 and is also made available for use under a CC0 license.

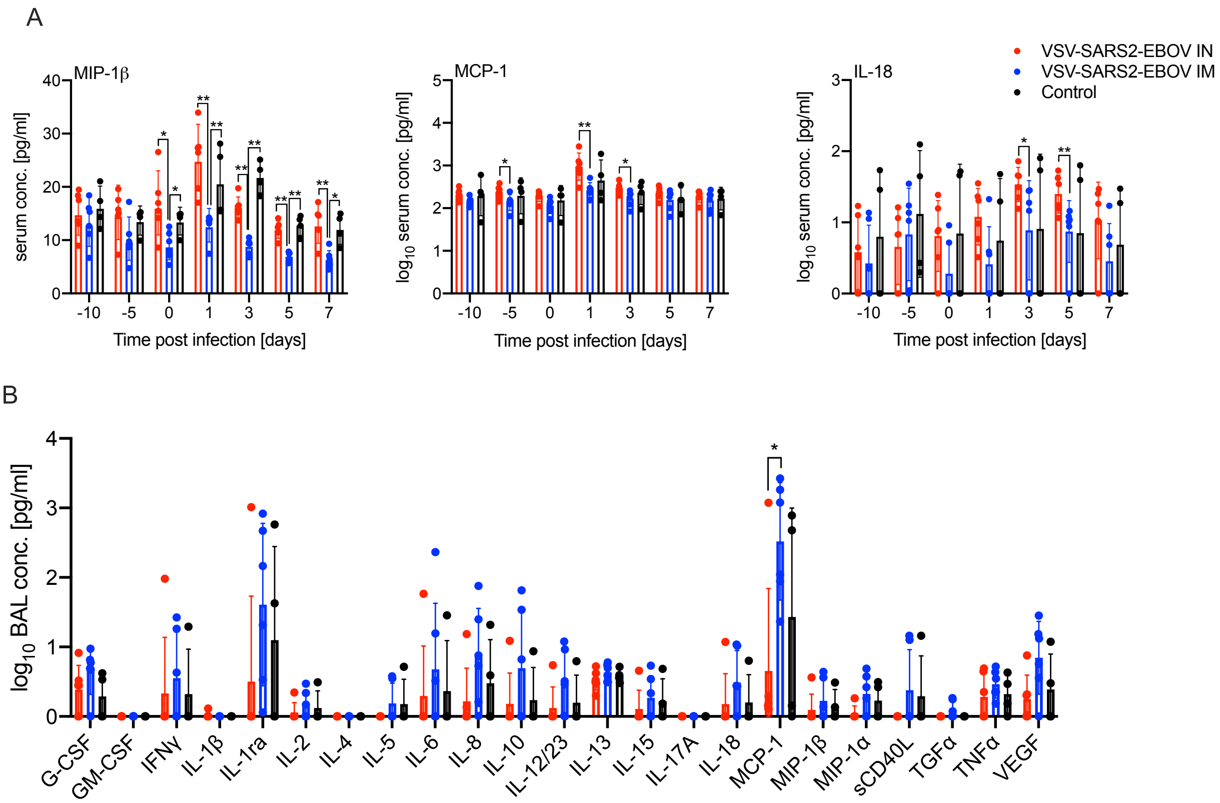


**Figure S5. Serum cytokine levels in NHPs after vaccination and challenge. (A)** Serum samples collected throughout the study and **(B)** bronchoaveolar lavage (BAL) samples collected on day 3 were analyzed. Statistical significance is indicated.

bioRxiv preprint doi: https://doi.org/10.1101/2021.01.19.426885; this version posted January 19, 2021. The copyright holder for this preprint (which was not certified by peer review) is the author/funder. This article is a US Government work. It is not subject to copyright under 17 USC 105 and is also made available for use under a CC0 license.

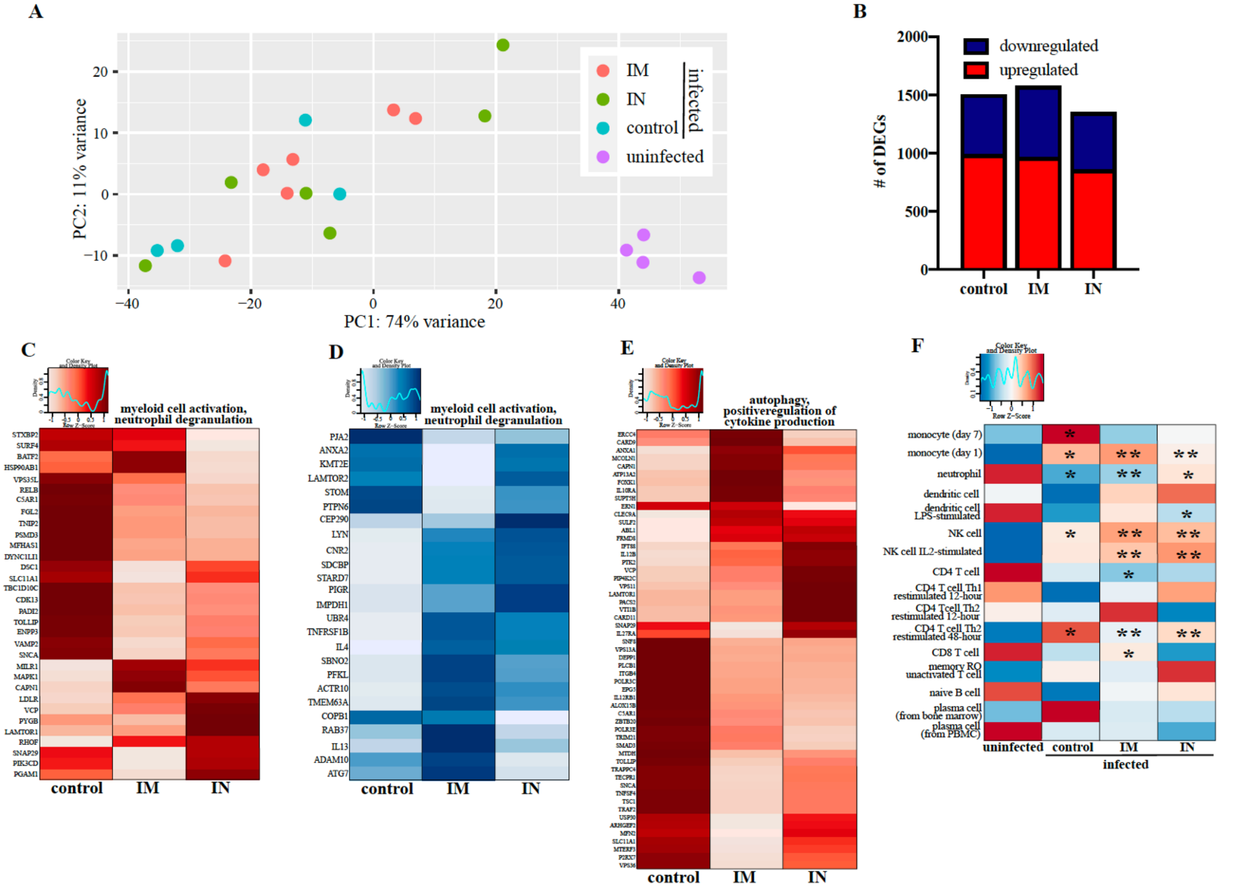


**Figure S6. BAL RNA-sequencing**. **(A)** Principal component analysis of bronchoalveolar lavage (BAL) samples from uninfected animals and vaccinated animals 3 days post challenge (control, intramuscular (IM) or intranasal (IN) vaccination). **(B)** Down- and up-regulated differentially expressed genes (DEGs). Heatmaps representing DEGs shared by all infected groups and enriching to Gene Ontology (GO) terms "myeloid cell activation" and "neutrophil degranulation" for **(C)** upregulated DEGs and **(D)** downregulated DEGs, and **(E)** "autophagy" and "positive regulation of cytokine production" for upregulated DEGs. Each column represents the median rpkm of the given group. Range of colors is based on scale and centered rpkm values of the represented DEGs. Red represents upregulated DEGs; blue represents downregulated DEGs. **(F)** *In silico* flow cytometry using ImmQuant IRIS database comparing challenged groups to uninfected controls. Red represents upregulation; blue represents downregulation. Each column represents the average relative predicted frequency of the given cell type. P-values are calculated relative to the uninfected animals. Statistical significance is indicated. For all heatmaps, range of colors is based on scale and centered rpkm values of the represented DEGs.

bioRxiv preprint doi: https://doi.org/10.1101/2021.01.19.426885; this version posted January 19, 2021. The copyright holder for this preprint (which was not certified by peer review) is the author/funder. This article is a US Government work. It is not subject to copyright under 17 USC 105 and is also made available for use under a CC0 license.

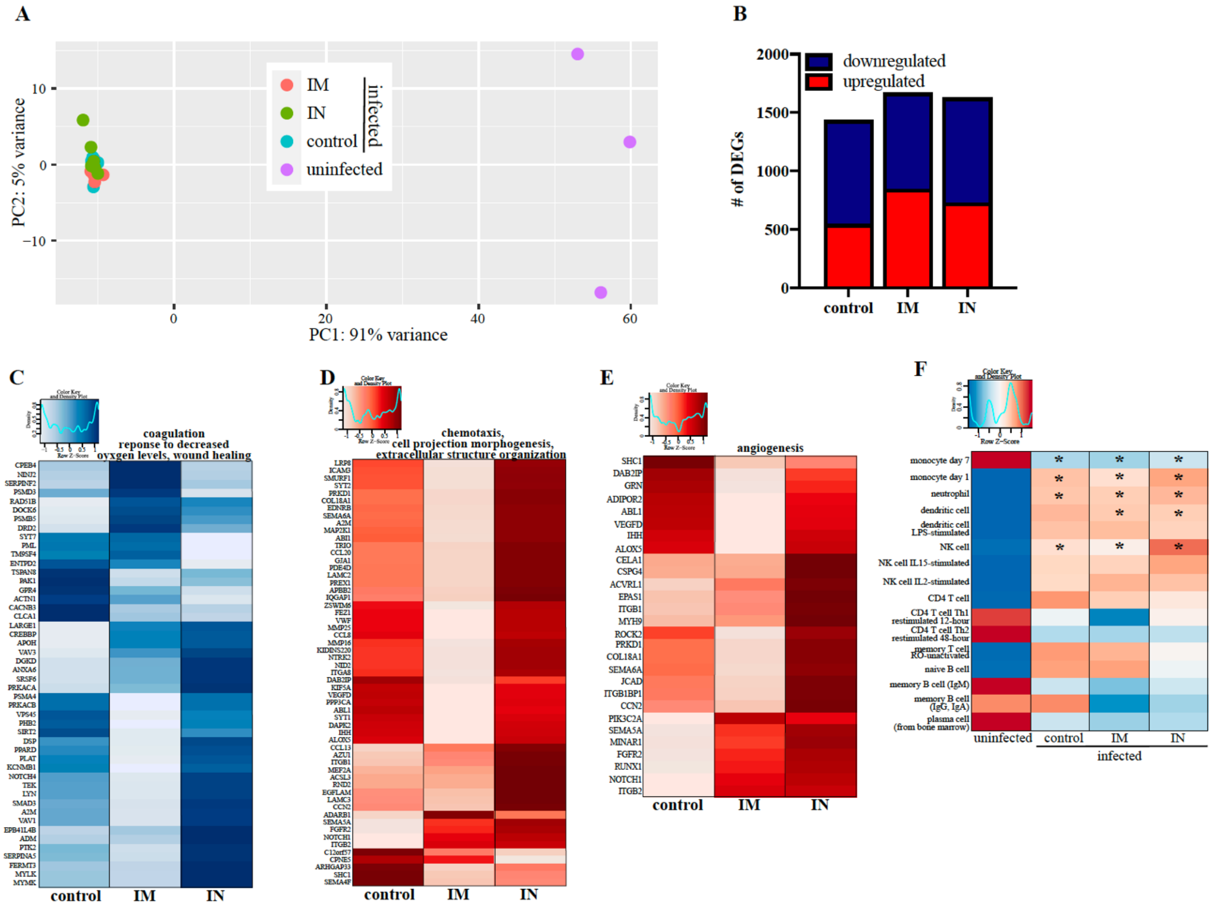


**Figure S7. Lung RNA-sequencing. (A)** Principal component analysis of lower left lung (LLL) samples from uninfected animals and vaccinated animals 7 days post challenge (control, intramuscular (IM) or intranasal (IN) vaccination). **(B)** Down- and up-regulated differentially expressed genes (DEGs). Heatmaps representing DEGs shared by all infected groups and enriching to Gene Ontology (GO) terms **(C)** “coagulation”, “response not decreased oxygen levels” and “wound healing” for downregulated DEG; and **(D)** “chemotaxis”, “cell projection morphogenesis” and “extracellular structure organization” and **(E)** “angiogenesis” for upregulated DEGs. Each column represents the median rpkm of the given group. Range of colors is based on scale and centered rpkm values of the represented DEGs. Red scale represents upregulated DEGs; blue scale represents downregulated DEGs. **(F)** *In silico* flow cytometry using ImmQuant IRIS database comparing challenged groups to uninfected controls. Red represents upregulation; blue represents downregulation. Each column represents the average relative predicted frequency of the given cell type. P-values are calculated relative to the uninfected animals. Statistical significance is indicated. For all heatmaps, range of colors is based on scale and centered rpkm values of the represented DEGs.
